# Oligodendroglial membrane dynamics in relation to myelin biogenesis

**DOI:** 10.1007/s00018-016-2228-8

**Published:** 2016-05-03

**Authors:** Hande Ozgen, Wia Baron, Dick Hoekstra, Nicoletta Kahya

**Affiliations:** Department of Cell Biology, University Medical Center Groningen, University of Groningen, Antonius Deusinglaan 1, 9713 AV Groningen, The Netherlands

**Keywords:** Oligodendrocytes, Myelin biogenesis, Fluorescence correlation spectroscopy, Membrane microdomains, Model membranes

## Abstract

In the central nervous system, oligodendrocytes synthesize a specialized membrane, the myelin membrane, which enwraps the axons in a multilamellar fashion to provide fast action potential conduction and to ensure axonal integrity. When compared to other membranes, the composition of myelin membranes is unique with its relatively high lipid to protein ratio. Their biogenesis is quite complex and requires a tight regulation of sequential events, which are deregulated in demyelinating diseases such as multiple sclerosis. To devise strategies for remedying such defects, it is crucial to understand molecular mechanisms that underlie myelin assembly and dynamics, including the ability of specific lipids to organize proteins and/or mediate protein–protein interactions in healthy versus diseased myelin membranes. The tight regulation of myelin membrane formation has been widely investigated with classical biochemical and cell biological techniques, both in vitro and in vivo. However, our knowledge about myelin membrane dynamics, such as membrane fluidity in conjunction with the movement/diffusion of proteins and lipids in the membrane and the specificity and role of distinct lipid–protein and protein–protein interactions, is limited. Here, we provide an overview of recent findings about the myelin structure in terms of myelin lipids, proteins and membrane microdomains. To give insight into myelin membrane dynamics, we will particularly highlight the application of model membranes and advanced biophysical techniques, i.e., approaches which clearly provide an added value to insight obtained by classical biochemical techniques.

## Introduction

In the central nervous system (CNS), processes protrude from oligodendrocytes (OLGs) at the end of which sheet-like extensions are formed, the myelin membranes, which ensheath axons in a multilamellar fashion to provide proper saltatory nerve conduction [1]. Myelin membranes are unique in that approx. 70 % of their dry weight consists of lipids, in particular cholesterol and the galactolipids galactosylceramide (GalC) and sulfatide (Table 1, [1, 2]). Myelin also contains a specific repertoire of myelin proteins, among which proteolipid protein (PLP) and myelin basic protein (MBP) are the most abundant ones [1–3]. The interactions between lipids and proteins are pivotal for myelin formation and maintenance, regulating protein transport to and the molecular organization within the myelin sheath [2, 4, 5]. Thus, biochemical and biophysical properties of the lipids actively control (myelin) protein sorting, while (myelin) proteins, in turn, are able to organize lipids, thereby creating regions of specialized molecular packing (e.g. lipid rafts) and dynamics, relevant to their functioning [6–8]. These membrane subdomains are usually enriched in cholesterol, glycosphingolipids and phospholipids with saturated fatty acids, providing a more ordered i.e., less dynamic microdomain organization (‘liquid ordered’) compared to the remainder of the membrane, which is more fluid or ‘liquid disordered’. Indeed, the ‘lipid raft’ concept is also very important for myelin research given that such rafts play a role in orchestrating signaling platforms that regulate OLG behavior, and sorting of several myelin proteins [2, 4, 6, 9, 10].

**Table 1:** Major myelin lipids

Lipid	% total dry weight of myelin			Comments	Reference	Notes
	Human	Bovine	Rat			
Cholesterol	27.7	28.1	27.3	Rate limiting for CNS myelination Insulator function of myelin	[173, 174]	
Galactolipids						
Galactosylceramide (GalC)	22.7	24	23.3	Role in OLG maturation Role in proper CNS node and paranode formation^a^ Role in electrophysioloigcal properties of myelin^a^ Stabilization and maintenance of axoglial adhesion at the paranode^a^ Maintenance of compact myelin^a^ Role in myelin integrity and stability^a^	[60, 65, 175–179]	Mainly C(24:1)
Sulfatide	3.8	3.6	7.1	Negative regulator of OLG differentiation Role in sodium channels clustering Role in paranode formation Role in NF155 organization	[63, 67, 180, 181]	Mainly C(24:1)

^a^The mentioned comment are obtained from CGT knock-out mouse studies, therefore these findings are also relevant for the function of sulfatide

Since all elements of the myelination machinery require a careful mutual orchestration, a defect in one of them could affect the entire machinery. Thus, alterations in protein–lipid trafficking or misfolding in conformational changes of myelin proteins may cause severe overall patho-neurological consequences. Therefore, insight into the spatio-temporal architecture of OLGs and their myelin membranes, the important players in myelin membranes is crucial to improve our understanding of the (re)myelination machinery, and hence the potential in developing (novel) therapeutic strategies. So far, the majority of studies in myelin membrane research have been performed with living cells. However, model membranes such as large unilamellar (LUVs) or giant unilamellar vesicles (GUVs) in conjunction with classical biophysical techniques are quite promising tools to investigate the lipid organization and domain assembly, as well as lipid–lipid and lipid–protein interactions [3, 11–15]. Additionally, single-molecule biophysical optical microscopy offers a set of highly sophisticated tools for gaining detailed molecular insight into the dynamic organization of lipids and proteins, which, most importantly, can be accomplished in a non-invasive manner, also in living cells. These approaches include photon counting histogram (PCH) analyses [16], F techniques [e.g., fluorescence recovery after photobleaching (FRAP) [17] and fluorescence correlation spectroscopy (FCS) [18], advanced FCS techniques (e.g. scanning [19], dual focus [20], *z*-scan [21], spot variation FCS [22]), fluorescence cross-correlation spectroscopy (FCCS) [23], and Förster resonance energy transfer (FRET) [24, 25]), image correlation techniques (e.g., raster image correlation spectroscopy (RICS) [26, 27], and number and brightness analyses (N&B) [28]). Here, we will summarize and discuss myelin assembly and maintenance in terms of the interaction of its structural elements, i.e., lipids, known to be enriched in the myelin membrane, and myelin-specific proteins. Additionally, we will highlight the versatility of biophysical technology in OLG-myelin research, and the impact of such approaches in improving our understanding of how lipids and proteins regulate the tight spatio-temporal organization of the myelin membrane and, thereby, its physiological function. Notably, a combined approach of highly advanced biophysical and classical biochemical techniques may not only clarify basic questions in myelin biology, a similar approach may also be most beneficial towards advancing our understanding of numerous other cell biologically relevant processes, ranging from vesiculation to membrane fusion and cellular secretion.

## Myelin biogenesis and structure: involvement of a set of specialized proteins and lipids

**Fig. 1:**
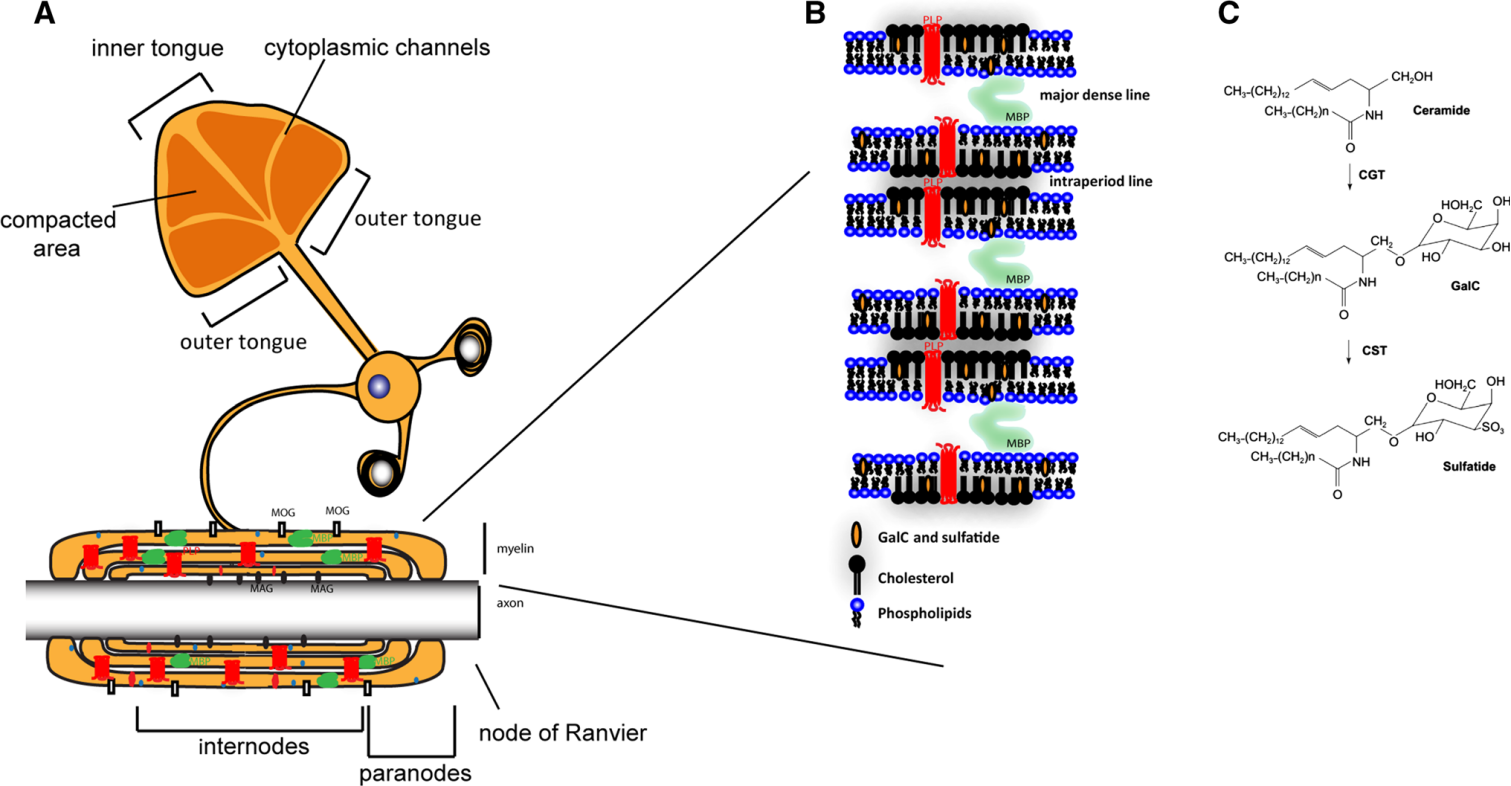
Myelin structure. **a** Schematic model that shows the uncompacted myelin sheath and the enwrapment of axons by myelin and the localization of major myelin proteins. The major myelin protein PLP is represented in red and MBP is represented in green. **b** Detailed schematic model of myelin membrane organization and the localization of the major myelin lipids GalC, sulfatide, cholesterol and myelin proteins PLP and MBP within the myelin membrane. Note that outerleaflet lipids GalC and sulfatide face each other in enwrapped myelin. **c** The synthesis scheme of sulfatide and GalC. Note that GalC is synthesized from ceramide by CGT (ceramide glucosyltransferase); sulfatide is subsequently synthesized from GalC by CST (cerebroside sulfotransferase)

The multilayered myelin membrane displays a distinct and complex architecture, i.e., in the process of enwrapping axons, the outer leaflets of the myelin membranes appose each other, thereby creating the intraperiod line, while the condensed cytoplasmic surface constitutes the major dense line ([1, 5], Fig. 1a, b), as readily visualized by electron microscopy (EM). The myelinated segments of the axons, the so-called internodes, are interchanged with myelin-devoid areas, named the ‘nodes of Ranvier’, where sodium channels are localized that generate a membrane potential that drives the action potential along the axon in a saltatory manner. Besides axonal enwrapping, myelin compaction takes place, which thus gives rise to areas of compact and non-compact myelin [1, 5]. Recent findings in rodents suggest that the compaction of the myelin sheath starts from the outer tongue and gradually shifts towards the inner tongue (leading edge of the sheath, Fig. 1a, [29]). Within the internodes different degrees of compaction can be discerned. Although mainly compact within the inter-node, regions at their edges, known as ‘paranodes’, consist of non-compact myelin (Fig. 1a). Interestingly, the molecular composition of compact and non-compact myelin differs; i.e., the major myelin proteins PLP and MBP together with the glycosphingolipid GalC reside in compact myelin, whereas other myelin proteins, such as 155-kDa neurofascin (NF155), together with the glycosphingolipid sulfatide localize in the paranodal region of non-compact myelin [2, 30]. The proper ‘compartmentalization’ of the myelin sheath accomplished in this manner is crucial for its function, because any alterations in the complex interrelated lipid and protein organization of myelin structure might cause more or less severe demyelinating, dysmyelinating, and hypomyelinating diseases. For example, in the hypomyelinating diseases Pelizaeus-Merzbacher disease (PMD) and the less severe spastic paraplegia 2 (SPG2) mutations, deletions or duplication of the PLP gene lead to abnormal myelin formation [31–34]. The mutations and deletions induce an alteration in the protein conformation, thereby perturbing PLP transport to the myelin sheath and affecting intracellular cholesterol/lipid transport to the myelin sheath [35, 36]. In the dysmyelinating disorder metachromatic leukodystrophy (MLD) [37], arylsulfatase A deficiency leads to accumulation of sulfatide in lysosomes. This causes a perturbation of the myelin structure, which is followed by demyelination, and a delay in remyelination. In other diseases, including multiple sclerosis (MS), myelin is initially formed in a correct manner, but severe myelin loss in the absence of remyelination results in persistent demyelination. [38]. The exact cause for demyelination in MS is still unknown, but in contrast to MLD a genetic origin is not likely. In MS, remyelination failure is likely due to an altered extracellular signaling microenvironment that among others affects the organization of OLG membranes, which causes defects in myelin at the molecular level [39–42].

For myelin biosynthesis to occur, progenitors cells of the myelin producing OLGs first have to mature to myelin-competent cells along a well-defined differentiation timeline [1, 43]. In this process, OLGs synthesize myelin specific proteins and lipids in a sequential and carefully regulated manner. Specifically, the myelin galactolipids GalC and sulfatide are produced prior to the expression of PLP and MBP. Therefore, the timing of the myelination machinery is crucial. In vivo, the generation of new myelin sheaths per individual OLG occurs in a relatively short and dynamic time window of approximately 5 h in zebra fish, after which maturation of the myelin sheaths requires another 1–2 days [44]. Also in rodents, a rather short time window of myelin sheath generation exists, e.g. co-cultured OLGs do not generate new myelin sheaths after 12 h [45]. Obviously, it takes much longer before the entire (re)myelination process of an entire axon is completed [46]. Thus, over a relatively short period of time myelin biogenesis may require vast amounts of building blocks to be produced within an OLG for sheath assembly. Major issues to be resolved in this regard are the identification and functioning of key players that orchestrate these events in a strictly temporal and spatial manner. Throughout myelin biogenesis and its maintenance, OLGs sort and transport different cargos from the cell body, via primary processes to the growing myelin sheath. In order to understand these events it has to be taken into account that OLGs display a polarized nature. Cell polarization is a well-known feature of epithelial and endothelial cells, consisting of a basolateral surface of a relative fluid nature and an apical surface, which is specifically enriched in glycosphingolipid and cholesterol, thus giving rise to a more rigid membrane domain. OLGs contain two distinct membrane surfaces, the plasma membrane surrounding the cell body, resembling the basolateral membrane in terms of composition, and the myelin membrane, which is reminiscent of an apical membrane domain in terms of composition. The presence of these distinct compartments might imply that polarized sorting and transport is likely instrumental in myelin biogenesis [2, 47]. Indeed, previous studies from our laboratory revealed the existence of polarized pathways in OLGs; the myelin sheet being targeted by a basolateral-like trafficking mechanism [2, 48], whereas transport of newly synthesized plasma membrane proteins reach this membrane via an apical-like trafficking mechanism. This shows that OLGs display a ‘unique’ polarity, where the cell body with the basolateral-like composition is targeted by an apical-like trafficking mechanism whereas the more apical-like myelin membrane is targeted by a basolateral-like mechanism. Consistently, the t-SNAREs syntaxin 3 and 4, which are distributed in a distinct polarized fashion in polarized cells, localize in OLGs to the plasma membrane and myelin membrane, respectively [2, 49]. Similarly, as reported for its transport in epithelial cells, the apical membrane protein marker hemagglutinin (HA) of influenza virus is delivered to the apical-like cell body plasma membrane in OLGs, while the VSV G protein, a basolateral marker in epithelial cells, is delivered to the myelin sheet [48]. Also, in vivo, VSV G is delivered to the inner tongue, i.e., the leading edge of the growing myelin membrane [29].

In more recent work evidence supports a transcytotic transport mechanism for PLP, indicating transport of de novo synthesized PLP from the endoplasmic reticulum to the myelin membrane via the OLG plasma membrane [50]. From the latter membrane, newly arrived PLP appears to be internalized via a sulfatide-mediated and clathrin-independent but cholesterol-dependent pathway [51], and it has been proposed that endosomal compartments may subsequently serve as site of storage, prior to a neuronal signal-triggered delivery of the protein to the myelin membrane, as inferred from data obtained in Oli-neu cells [51]. By contrast, the peripheral membrane protein MBP, another major myelin protein, is translated ‘on site’, following transport of MBP mRNA containing granules towards the myelin sheet [3, 49]. During the process of myelin wrapping around the axon, both PLP protein and MBP mRNA are continuously trafficking towards the myelin membrane via cytoplasmic channels near the non-compacted edges of the newly assembling myelin membrane (Fig. 1a, [52–54]). Furthermore, stimulation of myelin synthesis leads to an opening or closing of these cytoplasmic channels, which further emphasizes the dynamic structure of the myelin sheath [29].

Although considerable progress has been made in recent years in clarifying aspects of the underlying mechanism in myelin biogenesis in rodents, further improvement will require detailed insight into the fundamental role of myelin lipids and proteins in myelin assembly. We will therefore first focus on current knowledge of the involvement of the major myelin glycosphingolipids, i.e., the galactolipids GalC and sulfatide.

## Major myelin lipids galactosylceramide and sulfatide and their role in the myelin membrane

The lipid pool of myelin consists of phospholipids, plasmogens, cholesterol and glycosphingolipids [2, 10, 55, 56], and does not contain unique, i.e., myelin-specific lipids. Nevertheless, the glycosphingolipid GalC and its sulfated derivative, sulfatide (Fig. 1c) can be considered as ‘typical’ myelin lipids because of their relative high abundance, representing approx. 23 and 4 %, respectively, of the total lipid pool. These lipids, often referred to as galactolipids, are highly ordered lipids with long saturated and monosaturated fatty acyl chains, containing 22–26 carbon atoms [57]. Cholesterol is another abundant (approx. 28 % of the total lipid pool weight) and important structural lipid element of the myelin membrane (Table 1; [58, 59]), not in the least because of its ability to engage with the galactolipids in the formation of specific membrane microdomains, as will be discussed below.

Although galactolipids are important key players in safeguarding integrity and long term maintenance of myelin membranes, their presence does not seem to be essential for myelin biogenesis and assembly [60–64]. Based upon in vitro studies performed in rodents, it has been proposed that particularly GalC is important in OLG maturation [65], whereas sulfatide plays a role in OLG differentiation [66, 67]. However, more specific insight has been obtained in studies in which enzymes of galactolipid biosynthesis were downregulated, as in ceramide galactosyltransferase (CGT) knock-out mice, which are deficient in GalC, and consequently in sulfatide, for which GalC serves as a precursor (Fig. 1c, [64]). Even though myelin ultrastructure abnormalities are observed in this model, the biosynthesis of seemingly compacted myelin membranes does occur. However, upon closer examination, abnormal nodal and paranodal structures, a decrease in myelin stability over time and, importantly, disrupted axo-glia interactions are observed [62, 64]. This pathological phenotype of CGT null mice can be rescued by OLG-specific re-expression of CGT, which strongly supports the notion that the abnormalities observed in CGT null mice were indeed caused by GalC and/or sulfatide deficiency in OLGs [68]. Insight into a specific role of sulfatide could be obtained when the possibility was provided to create an animal model in which cerebroside sulfotransferase (CST), which is responsible for sulfatide synthesis from GalC, could be knocked-out [63]. Although the clinical phenotype was less severe, CST null mouse also revealed unstable myelin with age, disrupted paranodal compartments, axonal swellings and disruption in sodium channel clustering [10, 62]. Accordingly, these data evidently indicate that both GalC and sulfatide play distinct roles in OLG differentiation, myelin maintenance and overall stability and proper functioning of the myelin membrane.

## Major myelin proteins PLP and MBP and their role in the myelin membrane

Unlike its lipids, myelin expresses a unique set of proteins including PLP, MBP, myelin oligodendrocyte glycoprotein (MOG), myelin associated glycoprotein (MAG), 2′, 3′-cyclic-nucleotide 3′-phosphodiesterase (CNP) and NF155, of which PLP and MBP are the most abundant ones ([1, 2], Fig. 1a). Their participation in myelin biogenesis is a carefully regulated and timed process [1]. The integral membrane protein PLP with four membrane spanning domains consists of two different isoforms, i.e., PLP and DM20 [69]. PLP is expressed in late development, while DM20 is also expressed in embryonic or early development. Additionally, it has been shown in rats that PLP, mainly DM20, can be expressed by a specific set of neurons [70, 71]. PLP is synthesized at the endoplasmic reticulum, traffics through the Golgi apparatus, and is then transported in an indirect manner to the myelin membrane via a vesicular transport pathway [35, 50, 69, 72–74]. The protein primarily plays a role in stabilizing the intraperiod line by bringing together the extracellular leaflets of the myelin membrane (Fig. 1b), although PLP also participates in many other cellular processes, such as cholesterol transport and migration [74–77]. Nevertheless, PLP −/− mice show no dysmyelinating phenotype, indicating that PLP is not essential for the actual assembly of the myelin sheath. However, PLP might be important at the ultrastructural level once myelin has been formed, since in PLP −/− mice the extracellular compaction of adjacent membranes are abnormally condensed, which may be a sign of reduced myelin stability [78]. Interestingly though, as yet it cannot be excluded that another transmembrane protein with four membrane spans, M6B, can compensate for the lack of functional PLP in PLP knock-out animals, particularly since in case of a double knock-out of both PLP and M6B, severe hypomyelination is observed [74]. However, in a PLP deficient mice (PLP-neo mice), where an abnormal splice product of 159 is present, a disruption of the compacted myelin sheath was observed [79]. Also, overexpression of PLP and any mutation or deletion in the PLP gene causes abnormalities or severe pathological conditions in the context of gain of function, i.e., mutations of the PLP gene may cause an abnormal folding and hence perturbed conformational features of the protein, thereby dysregulating PLP trafficking, which results in extensive dysmyelination and/or premature OLG death [34–36, 80–83]. For example, duplication of PLP, as observed in PMD, results in accumulation of cholesterol and other raft components in an endosomal compartment. Accordingly, proper assembly of the myelin membrane fails [35, 84], which highlights the relevance of PLP’s presence in governing the proper assembly of cholesterol-containing membrane microdomains [74]. In addition, PLP/DM20 mutants have been reported to accumulate in a subset of neurons in the caudal brainstem of rats, which was accompanied by downregulation of the NMDA and GABA receptors [85]. Taken together, not the absence of PLP, but the expression of properly folded PLP at physiological levels appears to play a pivotal role in proper myelin assembly.

Different postnatal isoforms of the peripheral membrane protein MBP are expressed in a species-dependent manner, i.e., four isoforms have been discerned in rats, six in mice and four in humans. The MBP variants are produced from a single eleven exons containing gene complex (in mice), called Golli (‘gene in the OLG lineage’) [3, 86]. The classical MBP isoforms are derived from alternative splicing of a single MBP mRNA, which includes seven most downstream exons of the Golli gene complex. Among these isoforms, the ones containing exon-II, i.e., 17 and 21.5 kDa, shuttle between the cytoplasm and the nucleus, whereas the others, 14 and 18.5 kDa, localize mainly in compact myelin [3, 87, 88]. MBP is the only known structural myelin protein absolutely required for myelin membrane formation, presumably because of its role in myelin membrane compaction, due to its capacity of bringing the cytoplasmic leaflets together ([89, 90], Fig. 1b). Furthermore (exon-II minus) MBP is a multifunctional protein that has many different roles in signaling, cytoskeleton (actin, tubulin) polymerization and stability, and calcium-calmodulin binding [91–94]. In addition, it has been proposed that (exon-II minus) MBP serves as a molecular sieve, regulating the integration of proteins with a small cytoplasmic domain into the myelin membrane according to a mechanism that depends on MBP co-clustering in a condensed network, which triggers a phase transition in the myelin membrane and subsequently determines the exclusion or inclusion of other proteins into the myelin membrane [88, 95]. Because of its positive charge, MBP, as a cytoplasmic peripheral membrane protein, can interact with anionic phospholipids [e.g., phosphatidylserine (PS), phosphatidylinositol (PI)] in the inner leaflet of the myelin membrane [3]. Interestingly, MBP dynamics is also affected by changes of the dynamics of extracellular leaflet galactolipids [96, 97], suggesting that ‘indirect’ interactions may occur between the galactolipids, which constitute 60–70 % of the extracellular leaflet, and MBP, facing the cytoplasmic surface of the membrane. Moreover, by also taking into account its actin binding properties [91, 98], MBP might be a key player in transmitting galactolipid-derived signals.

## Membrane microdomains in oligodendroglial myelin

### Role of GalC and sulfatide

Apart from their presence or absence, the biochemical structure of galactolipids is another key factor in proper myelin functioning. Myelin membranes are mainly composed of galactolipids with very long saturated fatty acyl chains (C22-24) [99] and these galactolipid species, together with cholesterol and membrane proteins, are known to assemble into important and specialized membrane microdomains, so-called lipid rafts [100], operationally defined as detergent-insoluble or detergent-resistant membranes (for detailed reviews the reader is referred to [6, 7, 9]). Possibly, very long fatty acyl chain galactolipids are crucial for proper myelin maintenance and stability because they exert their function via these membrane microdomains. In this respect, it has been well established that membrane domain formation of the galactolipids is dependent on the chain length, their hydroxylation and saturation levels [101, 102]. Thus, hydroxylated or unsaturated lipids reveal a strongly diminished membrane microdomain forming capacity. For instance, the acyl chain length of sulfatide is developmentally regulated; i.e., prior to the onset of myelin formation (day 10 in rats), stearic acid (C18:0) is the main hydrocarbon chain present in sulfatide, whereas between day 10 and 32, nervonoyl sulfatide (C24:1) is upregulated [103]. Not only chain length but also hydroxylation of the fatty acyl chains of sulfatide is developmentally regulated; i.e., the extent of hydroxylation of sulfatides decreases with age in rats [103]. Therefore, it is also important to take into account the nature of the fatty acid chain and its state of hydroxylation when investigating and defining the specific role of galactolipids. Indeed, in an animal ceramide synthase 2 (CerS2) knock-out mice model, CerS2 being responsible for the synthesis of sphingolipids with very long (C22-24) fatty acid chains, a marked decrease in GalC and sulfatide levels was observed, while the phenotype showed unstable, and non-compacted myelin, with abnormalities in the inner lamellae [104]. Moreover, when reconstituted in a model membrane system, myelin lipid extracts obtained from CerS2 deficient mice, in contrast to such extracts from control animals, do not give rise to formation of membrane domains, [99]. Finally, with regard to hydroxylation, the hydroxylation levels of sulfatide are remarkably increased in MS patients. Since a decrease in hydroxylation promotes the formation of membrane microdomains in model membranes [102], these data suggest that such membrane microdomains in MS patients may be relatively decreased, which may have severe consequences for the proper assembly and lateral organization, and hence functioning (e.g. signaling), of the myelin membrane.

To appreciate the complexity of the myelin structure and organization, it is crucial to understand the ability of galactolipids to induce functional membrane microdomains and their role in membrane compaction. Such knowledge will require further insight as to how extracellular leaflet lipids transmit signals from and/or to the intracellular environment. For example, it has been proposed that galactolipids, given their relatively long fatty acyl chains, might interfere with the (lipid) organization in the inner leaflet as a result of acyl chain interdigitation, thereby ‘transmitting’ the signal. Consistent with this proposal, studies performed in model membranes indeed revealed that ceramide with C24:0 chains forms interdigitated gel phases [105]. Alternatively, it is also possible that galactolipid-mediated signal transmission proceeds via integral myelin membrane-specific proteins such as PLP, MAG or MOG. Consistent with such a possibility are observations that clustering of GalC in OLGs, occurring upon interaction of the cells with GalC-sulfatide liposomes, also causes clustering of the membrane spanning proteins PLP and MOG [106]. However, as yet, there is no direct evidence of a direct interaction between galactolipids and these integral membrane proteins, although such interactions might well be revealed by applying appropriate biophysical techniques or model membranes, as will be discussed in detail below (“Biophysical tools to investigate oligodendroglial-myelin membranes”).

### Myelin proteins and their membrane microdomain association

Membrane microdomains are composed of lipids and specific membrane proteins [9, 100, 107]. Hence, the partitioning behavior of integral or peripheral membrane proteins might have a pivotal effect on cellular activities dominated by lipid rafts, acting for example as signaling platforms. Several major and minor myelin membrane proteins are known to reside in membrane microdomains [6]. Operationally, these domains are often defined by their different detergent solubility behavior. For example, in myelin sheet-directed transport, following de novo biosynthesis, the major myelin protein PLP initially displays Triton-X100 (TX-100) insolubility, and acquires 3-[(3-cholamidopropyl)dimethylammonio]-1-propanesulfonate (CHAPS) insolubility at later steps in the transport pathway [2, 50, 106]. Remarkably, as a peripheral membrane protein, MBP also displays different detergent solubility behavior, as observed at different stages of OLG differentiation [108] and especially phosphorylated forms of MBP become CHAPS-insoluble at the myelin membrane [109]. Proteins such as NF155, MAL, and MOG display TX-100 insolubility or acquire TX-100 solubility by crosslinking (MOG); MAG, however, is Lubrol WX insoluble [6, 110], while NF155 and MAL in addition display Lubrol WX and CHAPS insolubility, respectively [30, 111]. The different detergent solubility properties might reflect the dynamic partitioning of the proteins in different membrane microdomains in OLGs and myelin.

It is still poorly understood whether myelin lipids represent the driving force in the lateral organization of myelin proteins or vice versa. In this regard, in differentiating OLGs, myelin galactolipids are expressed prior to myelin proteins [43]. Consistently, also our recent findings, derived from work in which conventional raft isolation procedures with detergent extraction were combined with optical biophysical techniques, revealed that sulfatide determines PLP’s partitioning into CHAPS-resistant membrane microdomains, whereas for MBP, the presence of GalC rather than sulfatide was pertinent for the protein’s partitioning in detergent-insoluble microdomains [50, 97]. In further support, several knock-out animal studies also suggest the importance of the membrane lipid composition in organizing the lateral distribution of myelin proteins into distinct membrane microdomains within the myelin sheath [73, 112]. Thus, in the CGT knock-out animal model the absence of galactolipids altered PLP’s association with CHAPS-insoluble membrane microdomans, i.e., under knock-out conditions PLP is largely CHAPS-soluble [73]. Similarly, inhibition of sphingolipid synthesis in primary OLGs disrupts the CHAPS-insolubility of PLP and MBP [73, 112]. However, in these studies it was also reported that in shiverer mice, where MBP is absent, a large fraction of PLP appears CHAPS-soluble, which could suggest that next to the presence of membrane galactolipids, the protein composition of the myelin membrane is also important for the lateral segregation of proteins [112]. However, in shiverer mice the galactolipid content decreases as well [90], implying that the membrane microdomain association of PLP might also be a consequence of an altered galactolipid content, which will require additional experimental work.

## Biophysical tools to investigate oligodendroglial-myelin membranes

### Model systems to study myelin lipid and proteins

#### Cell systems and model membranes

A need for further detailed mechanistic studies into the fundamental role of GalC and sulfatide in myelin biogenesis and maintenance is apparent from observations as reported above in knock-out animal models. For this purpose, one of the widely used models are rat or mouse OLG primary cell cultures [88, 95, 113]. These OLG primary cell cultures provide the possibility to study myelin membranes in vitro, since these cells, grown in monoculture, follow the same developmental pattern as in vivo. In fact, all the myelin components are expressed in a coordinated fashion and transported to the different subdomains in the myelin sheet, which are to a certain extent also compacted [88]. Moreover, the thinly flattened myelin-like sheets formed in monoculture, make them highly suitable for studies of intracellular trafficking, as compared to OLGs in vivo, where the myelin membrane is wrapped spirally around the myelinated axons. Hence, by taking into account the sequential expression of the galactolipids, primary cell cultures are an appropriate tool to tackle lipid-specific questions. Nevertheless, in these monocultures it is not possible to study the physiological complexity of the multi-layered myelin membranes, as in the case in the presence of the axons. For this purpose, the use of different culture systems in which OLGs and neurons are co-cultured can be applied to study myelin membranes while enwrapping axons [45, 114, 115]. However, with these in vitro models it is not possible to apply a reductionist approach, in which one can selectively express myelin lipids and proteins other than along its physiological expression timeline.

A convenient model for such reductionist studies appears to be OLG progenitor cell lines such as OLN-93 cells, which express neither GalC nor sulfatide [116]. However, in this rat-derived cell line, GalC or GalC and sulfatide can be readily and selectively expressed by cellular transfection with appropriate constructs of CGT, giving rise to the exclusive expression of GalC, and CGT/CST, which results in production of both GalC and sulfatide [97, 113]. However, an obvious limitation of this cell system is that the effect of sulfatide cannot be investigated as such, given that GalC serves as its precursor. Although addition of extracellular sulfatide to parental OLN-93 cells is an option, it is questionable whether exogenously inserted sulfatide will reside in the correct membrane domain. Alternatively, Oli-neu cells, an immature OLG cell line from mice, are also widely used in OLG-myelin research. However, these cells have the ‘disadvantage’ that they are not suitable for myelin lipid-dependent research, since they do express GalC and sulfatide, which is promoted upon differentiation by cAMP [117].

An attractive option to study specific myelin lipid-related questions is the application of simple membrane systems such as large unilamellar vesicles (LUVs), which may provide detailed insight into lipid–protein interactions between myelin lipids and protein [14], as will be discussed in the next section. An obvious choice would also be the use of model membranes such as giant unilamellar vesicles (GUVs). These model membranes are composed of lipids in which liquid ordered (Lo) and disordered (Ld) phases can coexist in a lipid composition dependent manner [118]. Thus, biological membranes have a special order and this order can be altered upon internal or external stimuli. For example, it is believed that the formation of so called ‘lipid rafts’ changes the membrane order and makes it tighter. In the case of model membranes like GUVs, it is also possible to create an order where some lipids can be tightly packed with different lipid compositions rather than having a homogenous lipid distribution. The liquid ordered phase displays a tight packing of lipids, similar as occurs in ‘lipid rafts’, whereas adjacent, less tightly packed lipids, the so called liquid disordered phase, displays a more fluidic environment (for further readings please see [11, 13, 119, 120]). In contrast to the relatively small LUVs, GUVs display diameters that may vary between 10 and 100 µm which makes them most convenient for visual inspection by optical microscopy. GUVs can be prepared from synthetic or natural lipids, allowing a large variation in fatty acyl chain length, hydroxylation levels, nature of the lipid head groups, and overall lipid composition [121, 122]. For example, GUVs reconstituted with GalC, sulfatide and glucosylceramide at similar ratios as present in myelin, revealed that depending on its concentration, glucosylceramide containing GUVs are fairly unstable (our unpublished observations), which might explain why in the absence of GalC and sulfatide, the upregulation of glucosylceramide synthesis cannot compensate for these galactolipids [123]. GUVs can also be prepared from natural lipid extracts [124, 125] or isolated native cell membranes [126]. In this manner, the lateral segregation of lipids extracted from different animal models, displaying different degrees in myelin perturbation, have been investigated. Thus, in GUVs prepared from myelin extracts obtained from MBP-deficient shiverer mice and CerS2-deficient mice, no membrane microdomain formation could be detected [99]. Nevertheless, domain formation in GUVs can be readily visualized using a fluorescent marker, which specifically attaches to the lipid of interest. For example, domain formation in GUVs, reconstituted with neuronal lipid extracts, was visualized with fluorescently-tagged cholera toxin, which specifically binds to ganglioside GM1, and with monoclonal antibody R24, which specifically binds to ganglioside GD3, to study the heterogeneity of the lipid rafts [127]. It is also possible to visualize phase separation behavior by using fluorescent lipid probes such as DIC, DIO, or DiD, which prefer partitioning in membrane domains, depending on their fluidity [128]. However, variability in results when applying these probes in detecting the liquid ordered or disordered phases have been reported, implying that care should be taken when interpreting the data. For example, DiIC18 showed preferential partitioning into the gel phases in GUVs, prepared from POPC (diacylglycerol and phospholipid)/sphingomyelin, whereas the probe partitioned into the fluid phase in GUVs prepared from POPC/DPPC (phospholipid) [128]. Moreover, although fluorescently labeled lipid analogues can be directly applied in the preparation of GUVs, it has also been reported that factors like the chemical structure of the fluorescent probe, the positioning of the fluorophore in the lipid analogue, the lipid composition of the bilayer, the chemical structure of the fatty acyl chain of the lipid analogue, and some thermodynamic properties such as the temperature, might change dramatically the domain formation behavior as such (reviewed in [129]).

The specific preference of myelin proteins for microdomains composed of distinct lipids can also be visualized in reconstituted model membrane systems (GUVs) by optical microscopy. Particularly as a myelin membrane model, it is relevant of being able to reconstitute GUVs selectively with either sulfatide or GalC. An important step of such procedures is of course the proper integration of the protein of interest in the reconstituted membrane. There are in fact several options to incorporate proteins into these model membranes [12, 130–132] and the preparation procedure depends on the nature of the protein.

#### Mimicking myelin membranes

To study the function of myelin membrane proteins or lipids can be troublesome with simple cell line systems or model membranes, because myelin membranes are the only membrane platform of which the extracellular leaflets of the same membrane are opposing each other, thereby giving rise to a multilamellar complex structure (Fig. 1b). Therefore, by using in vitro model systems consisting of a combined system of cells and opposing model membranes, the major dense line or intraperiod line can thus be mimicked, allowing a better understanding of the function of the major myelin proteins MBP and PLP, and myelin galactolipids.

Upon myelination, the sugar moieties of the myelin galactolipids, which exclusively localize at the extracellular leaflet in myelin, are facing each other. This fact prompted Boggs et al. to propose that myelin membranes might have a ‘glycosynapse’, where carbohydrate groups from opposing membranes are interacting [96, 106, 133]. Indeed, liposomes and nanoparticles, consisting of either GalC or both GalC and sulfatide, when incubated with primary OLGs, mimicking the intraperiod line as described above, are capable of inducing (1) a redistribution/clustering of GalC at the extracellular leaflet, (2) a reorganization of MBP at the cytoplasmic side of the membrane, and (3) a disruption of the actin cytoskeleton and tubulin network in OLGs [133, 134]. These fascinating observations highlight a potential role of galactolipids in creating signaling platforms, thereby regulating myelin compaction by perturbing MBP-membrane interaction and affecting cytoskeletal dynamics. In addition, galactolipids might act as their own receptors, receiving and/or transmitting signals via the glycosynapse in a density dependent manner [133]. Indeed, in this context, it has been recently demonstrated that the density and confluency of the coat of negatively charged oligosaccharides (i.e., the glycocalyx, in particular provided by the galactolipids in OLGs), may cause repulsion of the opposing membranes, and that this density-dependent electrostatic repulsion between opposing membranes is effectively downregulated upon differentiation of the OLG, i.e., conditions at which the myelin membrane is generated [135]. Furthermore, in the same study, the formation of the IPL was also mimicked by addition of liposomes or intraperiod line membranes from PLP knock-out mice. However, not unexpectedly, a proper interaction between the liposomes and the membranes of mature OLGs [135] was perturbed, supporting the role of PLP in the stabilization of intraperiod line and adhesion of extracellular leaflets of myelin to each other. Taken together, a partial loss of the glycocalyx might create an appropriate molecular and spatial environment for engagement of the galactolipids. This holds in particular for GalC as this lipid localizes in compact parts of the internodes, in (transient) interactions between opposing membranes in a PLP-dependent manner, thereby promoting myelin development and compaction.

Recently, it has been proposed that MBP is a major molecular factor in maintaining the high lipid to protein ratio in myelin by a mechanism in which it acts as a molecular sieve and diffusion barrier [88]. Next to the primary cell system, a biomimetic system was applied to study the physical barrier properties of MBP in which the apposition of the cytoplasmic part of the myelin membrane (major dense line) was mimicked by using supported bilayers (SPLSs), consisting of cytoplasmic leaflet lipids of myelin, and GUVs consisting of phosphatidylserine (PS) and phosphatidylcholine (PC). In this system, the distribution of a positively charged membrane-anchored GFP was monitored in the presence or absence of MBP. In the absence of MBP, membrane-anchored GFP, sandwiched between SPLs and GUVs, revealed a homogenous distribution whereas addition of MBP to this system caused a reallocation of GUVs to distinct areas on the SPLs, from which GFP was partially excluded. When MBP was sandwiched between SPLs and GUVs prior to addition of membrane-anchored GFP, a total exclusion of GFP occurred from the spread areas to which GUVs were attached. These data support the notion that MBP can also exert its physical barrier function in vitro systems, a conclusion that is most conveniently reached by employing a biomimetic system that allows freedom in the addition of relevant compounds. The sequential inclusion of MBP in this model system thus supports the protein’s ability to exclude homogenously distributed proteins from compacted sheaths.

### Measuring oligodendroglial-myelin membrane dynamics

In general, the molecular organization of the lipids within the membrane creates a special environment for the correct distribution of the protein and presumably, lipids and proteins may be considered as an ‘interactive team’, which determines the general structure and the assembly of the membranes. In the previous sections, we summarized findings indicative of the existence of lipid/protein cross-talk in OLGs and myelin membranes, largely inferred from biochemical and cell biological approaches. This cross-talk, as reflected by microdomains, or “rafts”, is also proposed to be very important for specific cellular functions because of their highly dynamic properties, i.e., they can be transiently formed upon external stimulation, which then might stimulate internal signaling, as occurs via the ‘glycosynapse’. However, when and how ‘rafts’ affect the dynamic properties of a membrane is still poorly understood. Therefore, a better understanding of these dynamics within the OLG cell body plasma membrane and myelin might shed light on crucial mechanisms, playing a role in the proper organization of the myelination machinery. Within this context, the application of advanced methodologies such as a combined approach of cell biology and biophysics might be most rewarding in improving our understanding (recent achievements are summarized in Table 2).

**Table 2:** Optical microscopical biophysical techniques applied in oligodendrocyte-myelin field in chronological order

References	Biophysical techniques	Major findings
[182]	FCS	Diffusion coefficient of MOG-eGFP and Bodipy FL-C5 sphingomyelin
[112]	C-Laurdan	In co-culture systems (neuron-derived signals): Increased membrane order in primary OLGs by neuron-derived soluble factors Increased membrane condensation in the presence of MBP
	FRAP	Formation of highly dynamic GalC clusters in OLGs
	STED	Increased number of large GalC clusters in the presence of neurons
	FRET	Self-interaction of GalC within the large clusters
[136]	FRET	Decrease in Rho activity in Oli-neu cells in the presence of neuronal-conditioned medium
	C-Laurdan	Increased membrane condensation in the presence of conditioned neuronal medium or RhoGTPase inactivation
[183]	RICS, FRAP	Diffusion coefficient of MOG-eGFP in OLN-93 cells
[164]	FRET	Interaction of 14-kDa MBP with PIP2 (sensed by CFP-PH-PLCδ1) in Oli-neu cells
[184]	RICS, FRAP	Diffusion coefficient of DiI-C18 in primary OLGs
[99]	C-Laurdan	Higher lipid order in GUVs prepared from myelin compared to Oli-neu cells Higher lipid order in GUVs prepared from wild type animal compared to shiverer mouse^a^ or CerS2-deficient mouse^b^ Higher lipid order in primary OLGs compared to FB1^c^-treated OLGs
	FCS	Slower diffusion of DiD in GUVs prepared from wild type animal compared shiverer^a^ mouse or CerS2 deficient mouse
	FRAP	Slower diffusion of Cell MaskOrange^d^ in control OLGs compared to FB1^c^-treated OLGs
[88]	FRAP	Detection of highly mobile MAG in OLGs isolated from shiverer mouse compared to control to show the diffusion barrier function of 14-kDa MBP Higher mobility of truncated Tmem10 (without cytosolic domain) compared to full length Tmem10
[87]	FRAP	Dynamic role of 21.5-kDa MBP-RFP in OLN-93 cells proliferation
[95]	FRAP	Decreased 14-kDa MBP diffusion due to its oligomerization
	FRET	Self association of 14-kDa MBP
[97]	s-FCS	Decreased mobility of PLP-eGFP in the presence of sulfatide (rather than GalC) on poly-l-lysine and laminin-2 in OLN-93 cells
		Increased mobility of PLP-eGFP even in the presence of sulfatide on fibronectin in OLN-93 cells
	*z*-scan FCS, *z*-scan RICS	Increased mobility of 18.5-MBP-eGFP in the presence of only GalC in OLN-93 cells
[166]	FRET	Presence of one or more intermediate conformational states of 18.5-kDa MBP

^a^MBP-deficient mouse

^b^Mouse model unable to synthesize long acid fatty acyl chain lipids

^c^Inhibitor of sphingolipid synthesis

^d^Membrane dye

#### Measuring membrane order (fluidity)

Membrane ordering/fluidity is determined by the presence or absence of membrane microdomains, and regulates membrane dynamics and rigidity and thereby the functioning of the membrane. Evidence is accumulating that myelin membrane order/fluidity is largely dependent on two major factors; firstly, the molecular composition of the myelin membrane; i.e., the abundance of long chain, microdomain forming galactolipids, and the presence of the peripheral membrane protein MBP. For instance, GUVs reconstituted with membrane extracts from CerS2-deficient mice devoid of long fatty acyl galactolipids, or from shiverer mice, devoid of MBP, revealed a decrease of membrane order compared to that of wild type animals [99]. In a similar way, the membrane fluidity is relatively increased in primary OLGs (1) derived from shiverer mice or (2) following fumonisin B1-mediated inhibition of sphingolipid synthesis. Secondly, myelin membrane fluidity is under tight control of neuron-derived soluble signals. The incubation of primary OLGs and Oli-neu cells with conditioned neuronal medium displayed increased membrane condensation [112, 136].

To determine the state of membrane ordering, fluorescent probes which specifically partition into liquid ordered or disordered phases are often used to investigate such domain formation in model membranes. However, the choice of probe in terms of obtaining unambiguous results can be rather challenging. For that reason, the application of different types of environmentally sensitive membrane dyes, such as di-4-ANEPPDHQ, Laurdan, PY3304, PY3174, and PY3184, is recommended [128, 137]. Particularly the use of Laurdan has greatly contributed to clarifying important biophysical issues in myelin membrane dynamics [138, 139]. This fluorescent probe is sensitive to membrane phase transitions and membrane fluidity. Thus the emission spectrum of membrane inserted Laurdan undergoes a blue shift from 500 to 430 nm, when the membrane domain in which the probe partitions undergoes a phase change from a liquid-disordered to liquid-ordered state. By monitoring, therefore, the fluorescence signal from two channels, it is possible to compose a generalized polarization (GP) image which provides the possibility to calculate the membrane order [128, 140]. The application of C-Laurdan, which has a greater membrane environment-dependent sensitivity and a diminished susceptibility towards photobleaching, further boosted its versatile use [139]. A particular advantage of this dye is that it homogenously distributes in the lateral plane of the membrane, while differences in membrane order are simply inferred from changes in its emission spectrum. Another advantage is that, (C-)Laurdan is suitable for use in both model and cell membranes.

Taken together, further applications of (C-)Laurdan can rule out other factors, i.e., soluble signals such as pro-inflammatory cytokines present in MS lesions or different extracellular matrix proteins, affecting the OLG-myelin membrane order and therefore dynamics. Moreover, the application of (C-)Laurdan to model membranes reconstituted from MS patient material or myelin lipids with modifications such as fatty acyl chain length, and hydroxylation (see above), can clarify the extent to which diseases such as MS can affect membrane order and how such changes may relate to physiological consequences. Notably, another approach, next to membrane order sensitive dyes, is Coherent anti-stokes Raman scattering (CARS) imaging. This particular technique does not require any labeling, since it determines the membrane order by relying on the molecular vibrations of the sample (for detailed information please read [141]). This technique can be even applied to live tissues. Also in the myelin field, CARS microscopy serves as an important tool to compare membrane order in healthy and diseased (e.g., EAE) animals [142, 143].

#### Measuring membrane dynamics

**Fig. 2:**
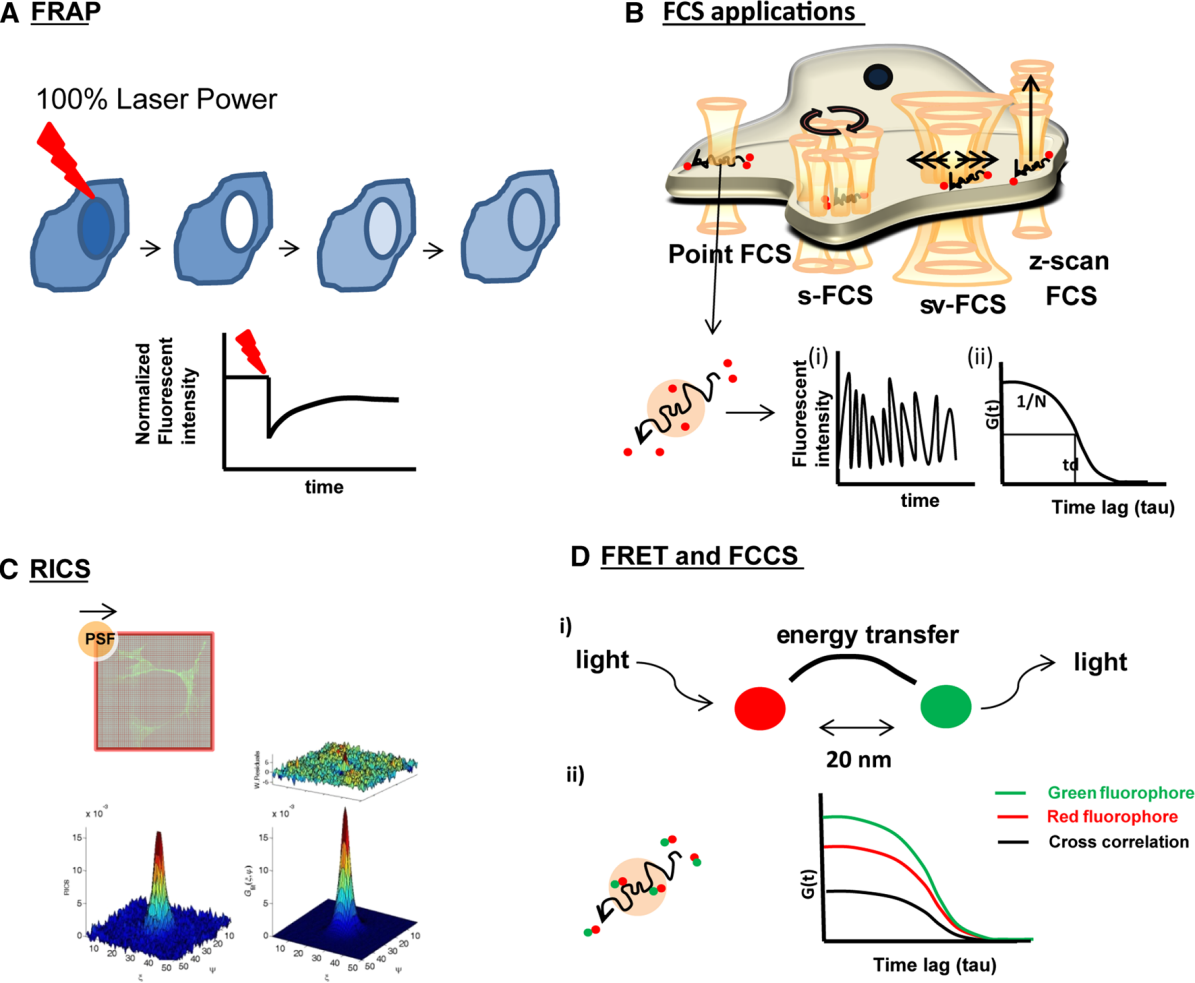
Biophysical applications. **a**. FRAP (fluorescence recovery after photobleaching) application in a living cell (for more details see the text). The laser beam depicted in red reflects 100 % laser power. The corresponding graph shows the fluorescence recovery after bleaching. **b** FCS (fluorescence correlation spectroscopy) applications in a living cell (for more details see the text). The laser beam is depicted in orange and the diffusing molecules in red. Fluorescently labeled molecules diffusing through the detection volume give rise to fluorescence fluctuations in time (*i*) which can be converted to the autocorrelation curve to determine the half decay. By fitting the autocorrelation curve with mathematical models, particle number, diffusion time/coefficient can be calculated (*ii*). **c** Schematic representation of RICS (raster image correlation spectroscopy). Temporal information can be extracted from raster scan images as these images are recorded pixel by pixel (for details see [26, 27]). A representative autocorrelation curve, the weighted residuals and corresponding 2D1C fit model is shown from a *z*-scan RICS measurement for 18.5 kDa MBP-eGFP. **d** (*i*) Schematic representation of FRET (fluorescence resonance energy transfer) and FCSS (fluorescence cross correlation spectroscopy). The *red* fluorophore is excited by laser light, which transfers its energy of the excited photon in a radiation-less manner to the *green* fluorophore which is thus excited and as a result emits light. For this so called principle of energy transfer, the distance between two fluorophores should be 20 nm or less. (*ii*) The *red and green* fluorophore diffuse together through the confocal volume (see **b**) which reveals cross correlation, depicted by the *black* cross-correlation curve in the corresponding graph

Even though membrane probes, such as (C-)Laurdan, or techniques such as CARS may provide insight into overall OLG-myelin membrane dynamics, such tools cannot extract dynamic information, originating from a specific lipid or protein, i.e., its lateral mobility. Therefore, to obtain more detailed information on the molecular dynamics, more advanced optical microscopic techniques in conjunction with sophisticated analyses are required, which can be applied to both living cells and model membranes. One such approach relies on FRAP, which is widely used to investigate molecular dynamics in biological systems, including myelin (recent findings are summarized in Table 2). FRAP allows quantification of dynamic information such as determination of the environment-dependent diffusion coefficient and the mobile fraction of the molecules of interest by applying a high laser power to bleach a region of interest (ROI), and recording the rate of fluorescence recovery (Fig. 2a). However, strictly speaking, FRAP cannot be considered as a non-invasive technique because of the usage of a high laser power to bleach fluorescently-labeled molecules in the ROI. In addition, FRAP cannot provide single molecule specificity, as is possible for other biophysical techniques such as fluorescent correlation spectroscopy (FCS). FCS is a powerful non-invasive biophysical technique, allowing determination of dynamical properties by applying very low laser light on a single point in the cell or at the cell’s surface, and recording the fluorescence fluctuations created by the diffusion of the fluorescently labeled molecules in and out the area of interest, which allows the determination of diffusion coefficients, and thereby the lateral mobility. However, given the complexity of the plasma and myelin membrane of OLGs, the application of more advanced approaches of FCS, in terms of acquired information and effectivity, are recommended (for a review see [144], Fig. 2b). Powerful options include scanning FCS, which provides the possibility to measure diffusion of the molecules simultaneously at different locations [145], and *z*-scan FCS, which provides the possibility to measure diffusion in different *z* planes, such as near the upper or bottom plasma membrane of plated cells [21].

Even though FCS has a high temporal resolution, it lacks spatial resolution, which may hamper determination of the membrane dynamics in cell types with complex morphology, like OLGs. Accordingly, image correlation techniques have been developed to deduce dynamic information from live cell systems; however, these techniques were not ideal because of their poor temporal resolution. A more recent technique, named raster image correlation spectroscopy (RICS) combined the temporal and spatial resolution in a confocal setup to make it more convenient for live cell systems. RICS can be applied with a conventional scanning confocal setup and allows for elimination of the immobile fractions from the obtained image and provides possibilities to select more precise spots within the ROI. However, restrictions are also readily apparent, in particular to apply RICS to very heterogeneously distributed proteins such as PLP, which localizes to a variety of transport vesicles, the cell body plasma membrane, the myelin membrane and, presumably, other intracellular (endosomal) membranes. In these kind of situations, it is more beneficial to apply biophysical techniques, such as circular scan FCS, where disruptions caused by vesicular structures can be eliminated (Fig. 2b, for further details please read [97]).

By application of techniques as described in the previous paragraph, evidence has been obtained that distinct myelin lipids act as key players in regulating the lateral mobility behavior of myelin proteins, and thereby OLG-myelin membrane dynamics. By using an FCS approach, Gielen et al. showed a membrane microdomain dependent change in the lateral mobility of BODIPY-labeled sphingomyelin in parental OLN-93 plasma membranes upon cholesterol depletion, a procedure that destroys membrane micodromain integrity. Additionally, we recently applied circular scan FCS to determine the dynamics of PLP-eGFP, and *z*-scan FCS and RICS to determine the dynamics of MBP-eGFP in relation to the myelin lipids GalC and sulfatide. The mobility of PLP decreased in the presence of sulfatide, which presumably reflects PLP-eGFP’s association with CHAPS-insoluble membrane microdomains under those conditions, as determined by classical biochemical means (detergent extraction). Additionally, the same study revealed that MBP dynamics was mainly governed by the presence of GalC, consistent with a potential signal transmission capacity between exoplasmic expressed GalC and MBP, which localizes at the cytoplasmic surface ([97], Table 2). Similarly, the lipid mobility measured by FCS in GUVs, reconstituted from myelin membranes of CerS2-deficient mice, devoid of long fatty acyl chain galactolipids and where membrane microdomain formation is not observed, was significantly faster than the rates determined in reconstituted membranes from healthy animals [99]. Hence, these findings provide new insight as to how the membrane microdomain forming nature of myelin (galacto)lipids might regulate the lateral mobility of myelin enriched lipids and specific proteins. In line with these findings, a combination of biochemical and biophysical studies suggested that the presence of the extracellular matrix protein fibronectin, a pathological condition which inhibits myelin membrane formation and impairs remyelination in MS lesions, might alter the membrane microdomain organization in OLG membranes by shifting sulfatide out of such domains [113]. As a consequence, the membrane microdomain association and the lateral mobility of PLP is likely affected [97]. These studies thus further suggest that the perturbation of the equilibrium of membrane microdomains in OLGs under diseased conditions may result in alterations in lateral mobility of myelin specific proteins. However, further investigations are needed to improve our understanding concerning the link between membrane microdomain association of a protein/lipid and its lateral mobility, which will be further discussed below.

#### Measuring membrane dynamics in relation to membrane microdomains

The mobility of microdomain associated fluorescently labeled lipid molecules has been extensively studied in a variety of model membranes by biophysical techniques such as FCS. In this manner it has been shown, for example, that in GUVs, the raft marker ganglioside GM1 diffuses relatively slowly, whereas the non-raft marker dialkylcarbocyanine dye diffuses considerably faster. This kind of studies support the notion that the dynamic behavior of a lipid is closely related to its microdomain association, i.e., microdomain associated lipids diffuse slower in the membrane. However, unlike in model membrane systems, where domain formation can be easily visualized due to their non-specific clustering, visualization of microdomains in native biological membranes is challenging given their size and life time. For example, in early work performed by FRAP analysis it was concluded that the lateral mobility of raft associated proteins is not necessarily slower than that of non-raft proteins [146]. On the other hand, in an elegant study relying on a sophisticated laser trap procedure, Pralle et al., demonstrated that when glycosylphosphatidylinositol (GPI) -anchored and transmembrane proteins are raft-associated, their diffusion becomes independent of the type of membrane anchor and is significantly reduced compared with that of non-raft transmembrane proteins [147].

Different approaches have been proposed in order to obtain a more direct clue on the dynamics of membrane proteins or lipids in relation to their membrane microdomain association. Marguet et al., proposed for the first time the spot variation FCS approach [22], based on the idea of the diffusion law, which was reviewed in detail in [8]. Briefly, in this method, the focal volume size where molecules pass through as a result of their diffusion properties is changed and the transient time that molecules spend in each focal volume is measured. Subsequently, the plotted transient time versus focal volume (focal spot area) gives three different diffusion patterns; free diffusion and hindered diffusion by either the cytoskeleton and/or a membrane microdomain (Fig. 2b). In that respect, spot variation FCS can be very informative for myelin research because it provides direct information about whether the lipids and proteins diffuse in a membrane microdomain or in a cytoskeleton-dependent manner. As an alternative to spot variation FCS, *z*-scan FCS makes it possible to determine the different diffusion types extracted by spot variation FCS in a commercial confocal setup [21]. In the *z*-scan, the diffusion time is plotted versus the ratio between the particle number at each *z* plane and the initial particle number. The first comparative study by *z*-scan FCS of the diffusion behavior of DiD in supported phospholipid bilayers (SPB) and OLN-93 cells, suggested that DiD diffuses freely in SPB while in OLN-93 cells the probe displays hindered diffusion due to the probe’s localization in rafts [21]. Therefore, an in-depth investigation about the type of diffusion behavior of myelin proteins or lipids, especially in primary OLGs, can provide further insight into the various diffusion types, such as hindered diffusion as a result of their association with microdomains and/or the cytoskeleton, which may differ at healthy versus pathological conditions.

### Lipid–protein and protein–protein interactions in oligodendroglial-myelin membranes

As evidenced by both biophysical and biochemical studies, the influence of myelin lipids on myelin proteins, for example in terms of trafficking and functioning, is of obvious relevance. However, the above mentioned studies do not provide direct information about the interactions between myelin lipids and proteins. To study such interactions, previous in vitro studies performed with artificial membranes such as LUVs with purified myelin proteins (especially MBP) provided detailed insight into myelin protein- inner leaflet lipid interaction as well as self-interaction of MBP [91, 148–153]. For instance, these studies revealed that MBP/inner leaflet lipid ratios in myelin membranes are quite crucial for MBPs ability to bind to negatively charged cytoplasmic leaflet lipids such as PS and PI; i.e., in the case of an MBP/lipid ratio, lower than that in compact myelin, MBP’s ability to induce aggregation of lipid bilayers decreases dramatically. Accordingly, since the concentration of the acidic phospholipid PS is dramatically increased in OLGs of MS patients, the MBP/lipid ratio is relatively decreased, which may thus lead to an impairment of MBP’s ability to mediate the adhesion of apposed cytoplasmic leaflets [154]. Additionally, studies performed with LUVs suggested that post-translational modifications of MBP such as deimination, which is dramatically increased in MS patients, might also change MBPs ability to adhere to the membrane [3, 155–157]. Since post-translational modifications of a protein may alter its structure and thereby its function dramatically, the effect of altered post-translational modifications of MBP can be studied at the structural by solid-state NMR [158]. Besides, the self-association of MBP near cytosolic leaflet lipids was shown to be non-random, but regular to some degree, by double electron–electron resonance (DEER) spectroscopy and solid-state NMR studies [148, 159]. In fact, these studies can be elaborated with more sensitive NMR technique called dynamic nuclear polarization (DNP) which is able to perform structural studies in biologically complex environments [160]. Moreover, a potential interaction of MBP with PIP(2), a minor component of the cytoplasmic leaflet of myelin, was first suggested with LUVs studies [161] and this data was further supported by showing the co-distribution MBP with PIP(2) sequestering proteins in primary rat OLGs by putative raft extraction [162].

Clearly, these kinds of approaches only provide detailed information at the model membrane level. At the cellular level, these interactions can be much more complex, especially when taking into account the presence of the extracellular leaflet lipids. In that sense, optical biophysical techniques such as FRET or fluorescence cross-correlation spectroscopy (Fig. 2d), where the measurements are done in live intact cells, can be very effective tools to investigate these interactions. The FRET technique relies on the principle of energy transfer between two chromophores, which depends on the distance between the molecules; i.e., if the distance between two molecules decreases then the efficiency of energy transfer increases (for details see [163], Fig. 2d). For example, by means of FRET, evidence was obtained showing that MBP and PIP2 interact at the plasma membrane of Oli-neu cells, providing the possibility to investigate the interactions of molecules within special compartments of the cells [164]. However, in some cases FRET cannot detect these interactions due to the distance limit. In those cases fluorescence cross-correlation spectroscopy might serve as a more informative tool because fluorescence cross-correlation spectroscopy determines whether two molecules are co-diffusing within the same confocal volume, independent of the distance [165, 23]. In that regard, it will be of interest to explore co-movement of major myelin proteins with (fluorescently labeled) lipids under different conditions, for example with or without the establishment of the ‘glycosynapse’, as discussed above. Alternatively, FRET can also be used to investigate the ‘self-interaction’ of the proteins. For instance, the self-association of MBP, and hence its oligomerization, has been revealed by a FRET study and the self-association of MBP on LUVs was also studied by a complementary biophysical technique, DEER [95, 148]. Moreover, a different approach for investigating the ‘self-interactions’ of proteins may also rely on specific techniques such as number and brightness analysis (N&B) or photon counting histogram (PCH) [16], which also allow determination of the degree of oligomerization. Such applications can provide further opportunities to investigate the potential oligomerization of PLP, which has been proposed to be regulated by sulfatide [50]. Also the conformational changes of PLP, can be investigated by FRET technique which has been applied to investigate conformational transitions of MBP [166].

Especially by considering the polarized nature of OLGs, these techniques, in conjunction with the unique opportunity of visualization by optical microscopy, can provide opportunities to specifically investigate ‘local’ lipid–protein and protein–protein interactions as well as protein oligomerization in the different regions (cell body and plasma membrane vs myelin sheath) in myelinating OLGs.

## Conclusion and outlook

Developing OLGs are exposed to many changes in their internal and external environment. Until the myelin sheath is synthesized and all the myelin-related components reach their proper destination, OLG-myelin membranes continuously undergo lateral reorganization. Hence, OLGs should display a very dynamic structure in order to regulate this series of events. Our knowledge about myelin biogenesis and OLG-myelin membrane dynamics is gradually improving. However, there are still numerous questions to be raised. For example, why does myelin display a unique galactolipid composition, and more specifically, why are membrane microdomain forming lipids highly enriched in myelin? How is this molecular organization formed and organized in space and time in the multi-lamellar compact myelin sheath? Do microdomain forming lipids have different role in compact and non-compact myelin? To what extent do membrane microdomains composed of galactolipids determine myelin membrane dynamics? In order to address and/or provide answers to these questions, necessary for revealing and clarifying vital internal mechanisms related to pathological demyelinating conditions, a detailed understanding of membrane dynamics of (human-derived) OLGs in the presence of axons and in myelin is needed. Most studies have been performed with rat or mouse OLGs, and validation and further studies in a human setting is an unmet need. Since it is rather difficult to culture primary human OLGs from post mortem tissue, the application of induced pluripotent stem cells may be the way to go, particularly since the composition of human myelin differs from that of rodent myelin [167]. Also, the lipid and protein composition of myelin, the latter also at the post-translational level, differ in MS lesions as compared to healthy brain [168, 169]. To visualize the distribution of posttranslational modified protein, imaging mass spectrometry (IMS), a rapidly emerging technique that allows for spatial profiling of post translational modified proteins in biological tissues and single cells, may be applied [170]. In fact, with this technique the in situ distribution of palmitoylated PLP and DM20 in rat brain was shown [171]. However this will not resolve the question whether the change in composition is a cause or consequence in MS. The composition of newly formed myelin after remyelination per se as compared to myelin formed upon development may differ, rather than that the disease alters the composition. On the other hand, given the altered myelin composition in normal appearing white matter [172], disease-specific extrinsic, but also OPC-related intrinsic factors, may contribute to the altered lipid and posttranslational signature of MS myelin. Also here patient-derived induced pluripotent stem cells are an interesting option to pursue on.

Here, we have summarized the role of myelin proteins and lipids in myelin biogenesis and introduced different models to study myelin biogenesis-related questions, and have illustrated how biophysical techniques can be exploited for this purpose (Table 2). For instance, optical microscopic applications, next to conventional biochemical techniques, already served as very informative tools to investigate the sophisticated biology of OLGs and myelin membranes. In addition, optical biophysical techniques prove to represent powerful, versatile tools in the analyses of protein and lipid dynamics in living OLGs in a non-invasive way.

## References

[1] Baumann N, Pham-Dinh D (2001). Biology of oligodendrocyte and myelin in the mammalian central nervous system. Physiol Rev.

[2] Baron W, Hoekstra D (2010). On the biogenesis of myelin membranes: sorting, trafficking and cell polarity. FEBS Lett.

[3] Boggs JM (2006). Myelin basic protein: a multifunctional protein. Cell Mol Life Sci.

[4] Krämer EM, Schardt A, Nave KA (2001). Membrane traffic in myelinating oligodendrocytes. Microsc Res Tech.

[5] Aggarwal S, Yurlova L, Simons M (2011). Central nervous system myelin: structure, synthesis and assembly. Trends Cell Biol.

[6] Gielen E, Baron W, Vandeven M (2006). Rafts in oligodendrocytes: evidence and structure-function relationship. Glia.

[7] Füllekrug J, Simons K (2004). Lipid rafts and apical membrane traffic. Ann N Y Acad Sci.

[8] He H-T, Marguet D (2011). Detecting nanodomains in living cell membrane by fluorescence correlation spectroscopy. Annu Rev Phys Chem.

[9] Dupree JL, Pomicter AD (2010). Myelin, DIGs, and membrane rafts in the central nervous system. Prostaglandins Other Lipid Mediat.

[10] Jackman N, Ishii A, Bansal R (2009). Oligodendrocyte development and myelin biogenesis: parsing out the roles of glycosphingolipids. Physiology (Bethesda).

[11] Bacia K, Schuette CG, Kahya N (2004). SNAREs prefer liquid-disordered over “raft” (liquid-ordered) domains when reconstituted into giant unilamellar vesicles. J Biol Chem.

[12] Kahya N, Brown DA, Schwille P (2005). Raft partitioning and dynamic behavior of human placental alkaline phosphatase in giant unilamellar vesicles. Biochemistry.

[13] Kahya N (2010). Protein–protein and protein–lipid interactions in domain-assembly: lessons from giant unilamellar vesicles. Biochim Biophys Acta.

[14] Hope MJ, Bally MB, Webb G, Cullis PR (1985). Production of large unilamellar vesicles by a rapid extrusion procedure: characterization of size distribution, trapped volume and ability to maintain a membrane potential. Biochim Biophys Acta.

[15] Vassall KA, Bamm VV, Harauz G (2015). MyelStones: the executive roles of myelin basic protein in myelin assembly and destabilization in multiple sclerosis. Biochem J.

[16] Chen Y, Müller JD, So PT, Gratton E (1999). The photon counting histogram in fluorescence fluctuation spectroscopy. Biophys J.

[17] Axelrod D, Koppel DE, Schlessinger J (1976). Mobility measurement by analysis of fluorescence photobleaching recovery kinetics. Biophys J.

[18] Magde D, Elson E, Webb WW (1972). Thermodynamic fluctuations in a reacting system—measurement by fluorescence correlation spectroscopy. Phys Rev Lett.

[19] Petersen NO (1986). Scanning fluorescence correlation spectroscopy. I. Theory and simulation of aggregation measurements. Biophys J.

[20] Dertinger T, Loman A, Ewers B (2008). The optics and performance of dual-focus fluorescence correlation spectroscopy. Opt Express.

[21] Humpolickova J, Gielen E, Benda A (2006). Probing diffusion laws within cellular membranes by *z*-scan fluorescence correlation spectroscopy. Biophys J.

[22] Wawrezinieck L, Rigneault H, Marguet D, Lenne P-F (2005). Fluorescence correlation spectroscopy diffusion laws to probe the submicron cell membrane organization. Biophys J.

[23] Schwille P, Meyer-Almes FJ, Rigler R (1997). Dual-color fluorescence cross-correlation spectroscopy for multicomponent diffusional analysis in solution. Biophys J.

[24] Piston DW, Kremers G-J (2007). Fluorescent protein FRET: the good, the bad and the ugly. Trends Biochem Sci.

[25] Selvin PR (1995). Fluorescence resonance energy transfer. Meth Enzymol.

[26] Digman MA, Brown CM, Sengupta P (2005). Measuring fast dynamics in solutions and cells with a laser scanning microscope. Biophys J.

[27] Digman MA, Sengupta P, Wiseman PW (2005). Fluctuation correlation spectroscopy with a laser-scanning microscope: exploiting the hidden time structure. Biophys J.

[28] Digman MA, Dalal R, Horwitz AF, Gratton E (2008). Mapping the number of molecules and brightness in the laser scanning microscope. Biophys J.

[29] Snaidero N, Möbius W, Czopka T (2014). Myelin membrane wrapping of CNS axons by PI(3,4,5)P3-dependent polarized growth at the inner tongue. Cell.

[30] Maier O, van der Heide T, Johnson R (2006). The function of neurofascin155 in oligodendrocytes is regulated by metalloprotease-mediated cleavage and ectodomain shedding. Exp Cell Res.

[31] Inoue K (2005). PLP1-related inherited dysmyelinating disorders: Pelizaeus-Merzbacher disease and spastic paraplegia type 2. Neurogenetics.

[32] Inoue K, Osaka H, Sugiyama N (1996). A duplicated PLP gene causing Pelizaeus-Merzbacher disease detected by comparative multiplex PCR. Am J Hum Genet.

[33] Koeppen AH, Robitaille Y (2002). Pelizaeus-Merzbacher disease. J Neuropathol Exp Neurol.

[34] Krämer-Albers E-M, Gehrig-Burger K, Thiele C (2006). Perturbed interactions of mutant proteolipid protein/DM20 with cholesterol and lipid rafts in oligodendroglia: implications for dysmyelination in spastic paraplegia. J Neurosci.

[35] Simons M, Kramer E-M, Macchi P (2002). Overexpression of the myelin proteolipid protein leads to accumulation of cholesterol and proteolipid protein in endosomes/lysosomes: implications for Pelizaeus-Merzbacher disease. J Cell Biol.

[36] Jung M, Sommer I, Schachner M, Nave KA (1996). Monoclonal antibody O10 defines a conformationally sensitive cell-surface epitope of proteolipid protein (PLP): evidence that PLP misfolding underlies dysmyelination in mutant mice. J Neurosci.

[37] Gieselmann V (2008). Metachromatic leukodystrophy: genetics, pathogenesis and therapeutic options. Acta Paediatr.

[38] Podbielska M, Banik NL, Kurowska E, Hogan EL (2013). Myelin recovery in multiple sclerosis: the challenge of remyelination. Brain Sci.

[39] Stoffels JMJ, de Jonge JC, Stancic M (2013). Fibronectin aggregation in multiple sclerosis lesions impairs remyelination. Brain.

[40] Colognato H, Tzvetanova ID (2011). Glia unglued: how signals from the extracellular matrix regulate the development of myelinating glia. Dev Neurobiol.

[41] Sobel RA, Ahmed AS (2001). White matter extracellular matrix chondroitin sulfate/dermatan sulfate proteoglycans in multiple sclerosis. J Neuropathol Exp Neurol.

[42] Back SA, Tuohy TMF, Chen H (2005). Hyaluronan accumulates in demyelinated lesions and inhibits oligodendrocyte progenitor maturation. Nat Med.

[43] Pfeiffer SE, Warrington AE, Bansal R (1993). The oligodendrocyte and its many cellular processes. Trends Cell Biol.

[44] Czopka T, Ffrench-Constant C, Lyons DA (2013). Individual oligodendrocytes have only a few hours in which to generate new myelin sheaths in vivo. Dev Cell.

[45] Watkins TA, Emery B, Mulinyawe S, Barres BA (2008). Distinct stages of myelination regulated by gamma-secretase and astrocytes in a rapidly myelinating CNS coculture system. Neuron.

[46] Czopka T (2016). Insights into mechanisms of central nervous system myelination using zebrafish. Glia.

[47] Simons M, Snaidero N, Aggarwal S (2012). Cell polarity in myelinating glia: from membrane flow to diffusion barriers. Biochim Biophys Acta.

[48] De Vries H, Schrage C, Hoekstra D (1998). An apical-type trafficking pathway is present in cultured oligodendrocytes but the sphingolipid-enriched myelin membrane is the target of a basolateral-type pathway. Mol Biol Cell.

[49] Bijlard M, Klunder B, de Jonge JC (2015). Transcriptional expression of myelin basic protein in oligodendrocytes depends on functional syntaxin 4: a potential correlation with autocrine signaling. Mol Cell Biol.

[50] Baron W, Ozgen H, Klunder B (2015). The major myelin-resident protein PLP is transported to myelin membranes via a transcytotic mechanism: involvement of sulfatide. Mol Cell Biol.

[51] Trajkovic K, Dhaunchak AS, Goncalves JT (2006). Neuron to glia signaling triggers myelin membrane exocytosis from endosomal storage sites. J Cell Biol.

[52] White R, Krämer-Albers E-M (2014). Axon-glia interaction and membrane traffic in myelin formation. Front Cell Neurosci.

[53] Paz Soldán MM, Pirko I (2012). Biogenesis and significance of central nervous system myelin. Semin Neurol.

[54] Nave K-A, Werner HB (2014). Myelination of the nervous system: mechanisms and functions. Annu Rev Cell Dev Biol.

[55] Schmitt S, Castelvetri LC, Simons M (2015). Metabolism and functions of lipids in myelin. Biochim Biophys Acta.

[56] Saher G, Stumpf SK (2015). Cholesterol in myelin biogenesis and hypomyelinating disorders. Biochim Biophys Acta.

[57] Lahiri S, Futerman AH (2007). The metabolism and function of sphingolipids and glycosphingolipids. Cell Mol Life Sci.

[58] Saher G, Simons M, Harris JR (2010). Cholesterol and myelin biogenesis. Cholesterol binding and cholesterol transport proteins.

[59] Saher G, Quintes S, Nave K-A (2011). Cholesterol: a novel regulatory role in myelin formation. Neuroscientist.

[60] Coetzee T, Fujita N, Dupree J (1996). Myelination in the absence of galactocerebroside and sulfatide: normal structure with abnormal function and regional instability. Cell.

[61] Marcus J, Honigbaum S, Shroff S (2006). Sulfatide is essential for the maintenance of CNS myelin and axon structure. Glia.

[62] Marcus J, Popko B (2003). Galactolipids are molecular determinants of myelin development and axo-glial organization. Biochim Biophys Acta.

[63] Honke K, Hirahara Y, Dupree J (2002). Paranodal junction formation and spermatogenesis require sulfoglycolipids. Proc Natl Acad Sci USA.

[64] Bosio A, Binczek E, Haupt WF, Stoffel W (1998). Composition and biophysical properties of myelin lipid define the neurological defects in galactocerebroside- and sulfatide-deficient mice. J Neurochem.

[65] Dupree JL, Suzuki K, Popko B (1998). Galactolipids in the formation and function of the myelin sheath. Microsc Res Tech.

[66] Hirahara Y, Bansal R, Honke K (2004). Sulfatide is a negative regulator of oligodendrocyte differentiation: development in sulfatide-null mice. Glia.

[67] Bansal R, Winkler S, Bheddah S (1999). Negative regulation of oligodendrocyte differentiation by galactosphingolipids. J Neurosci.

[68] Zöller I, Büssow H, Gieselmann V, Eckhardt M (2005). Oligodendrocyte-specific ceramide galactosyltransferase (CGT) expression phenotypically rescues CGT-deficient mice and demonstrates that CGT activity does not limit brain galactosylceramide level. Glia.

[69] Weimbs T, Stoffel W (1992). Proteolipid protein (PLP) of CNS myelin: positions of free, disulfide-bonded, and fatty acid thioester-linked cysteine residues and implications for the membrane topology of PLP. Biochemistry.

[70] Timsit S, Martinez S, Allinquant B (1995). Oligodendrocytes originate in a restricted zone of the embryonic ventral neural tube defined by DM-20 mRNA expression. J Neurosci.

[71] Michalski J-P, Anderson C, Beauvais A (2011). The proteolipid protein promoter drives expression outside of the oligodendrocyte lineage during embryonic and early postnatal development. PLoS ONE.

[72] Brown MC, Besio Moreno M, Bongarzone ER (1993). Vesicular transport of myelin proteolipid and cerebroside sulfates to the myelin membrane. J Neurosci Res.

[73] Simons M, Krämer EM, Thiele C (2000). Assembly of myelin by association of proteolipid protein with cholesterol- and galactosylceramide-rich membrane domains. J Cell Biol.

[74] Werner HB, Krämer-Albers E-M, Strenzke N (2013). A critical role for the cholesterol-associated proteolipids PLP and M6B in myelination of the central nervous system. Glia.

[75] Gudz TI, Schneider TE, Haas TA, Macklin WB (2002). Myelin proteolipid protein forms a complex with integrins and may participate in integrin receptor signaling in oligodendrocytes. J Neurosci.

[76] Gudz TI, Komuro H, Macklin WB (2006). Glutamate stimulates oligodendrocyte progenitor migration mediated via an alphav integrin/myelin proteolipid protein complex. J Neurosci.

[77] Greer JM, Lees MB (2002). Myelin proteolipid protein—the first 50 years. Int J Biochem Cell Biol.

[78] Klugmann M, Schwab MH, Pühlhofer A (1997). Assembly of CNS myelin in the absence of proteolipid protein. Neuron.

[79] Boison D, Stoffel W (1994). Disruption of the compacted myelin sheath of axons of the central nervous system in proteolipid protein-deficient mice. Proc Natl Acad Sci USA.

[80] Knapp PE, Skoff RP, Redstone DW (1986). Oligodendroglial cell death in jimpy mice: an explanation for the myelin deficit. J Neurosci.

[81] Skoff RP (1995). Programmed cell death in the dysmyelinating mutants. Brain Pathol.

[82] Gow A, Friedrich VL, Lazzarini RA (1994). Many naturally occurring mutations of myelin proteolipid protein impair its intracellular transport. J Neurosci Res.

[83] Gow A, Southwood CM, Lazzarini RA (1998). Disrupted proteolipid protein trafficking results in oligodendrocyte apoptosis in an animal model of Pelizaeus-Merzbacher disease. J Cell Biol.

[84] Saher G, Rudolphi F, Corthals K (2012). Therapy of Pelizaeus-Merzbacher disease in mice by feeding a cholesterol-enriched diet. Nat Med.

[85] Miller MJ, Haxhiu MA, Georgiadis P (2003). Proteolipid protein gene mutation induces altered ventilatory response to hypoxia in the myelin-deficient rat. J Neurosci.

[86] Harauz G, Boggs JM (2013). Myelin management by the 18.5-kDa and 21.5-kDa classic myelin basic protein isoforms. J Neurochem.

[87] Ozgen H, Kahya N, de Jonge JC (2013). Regulation of cell proliferation by nucleocytoplasmic dynamics of postnatal and embryonic exon-II-containing MBP isoforms. Biochim Biophys Acta.

[88] Aggarwal S, Yurlova L, Snaidero N (2011). A size barrier limits protein diffusion at the cell surface to generate lipid-rich myelin-membrane sheets. Dev Cell.

[89] Chernoff GF (1981). Shiverer: an autosomal recessive mutant mouse with myelin deficiency. J Hered.

[90] Readhead C, Hood L (1990). The dysmyelinating mouse mutations shiverer (shi) and myelin deficient (shimld). Behav Genet.

[91] Boggs JM, Rangaraj G, Heng Y-M (2011). Myelin basic protein binds microtubules to a membrane surface and to actin filaments in vitro: effect of phosphorylation and deimination. Biochim Biophys Acta.

[92] Smith G, Homchaudhuri L, Boggs J, Harauz G (2012). Classic 18.5- and 21.5-kDa Myelin basic protein isoforms associate with cytoskeletal and SH3-domain proteins in the immortalized N19-oligodendroglial cell line stimulated by phorbol ester and IGF-1. Neurochem Res.

[93] Smith GST, De Avila M, Paez PM (2012). Proline substitutions and threonine pseudophosphorylation of the SH3 ligand of 18.5-kDa myelin basic protein decrease its affinity for the Fyn-SH3 domain and alter process development and protein localization in oligodendrocytes. J Neurosci Res.

[94] Smith GST, Paez PM, Spreuer V (2011). Classical 18.5-and 21.5-kDa isoforms of myelin basic protein inhibit calcium influx into oligodendroglial cells, in contrast to golli isoforms. J Neurosci Res.

[95] Aggarwal S, Snaidero N, Pähler G (2013). Myelin membrane assembly is driven by a phase transition of myelin basic proteins into a cohesive protein meshwork. PLoS Biol.

[96] Boggs JM, Gao W, Zhao J (2010). Participation of galactosylceramide and sulfatide in glycosynapses between oligodendrocyte or myelin membranes. FEBS Lett.

[97] Ozgen H, Schrimpf W, Hendrix J (2014). The lateral membrane organization and dynamics of myelin proteins PLP and MBP are dictated by distinct galactolipids and the extracellular matrix. PLoS ONE.

[98] Harauz G, Ladizhansky V, Boggs JM (2009). Structural polymorphism and multifunctionality of myelin basic protein. Biochemistry.

[99] Yurlova L, Kahya N, Aggarwal S (2011). Self-segregation of myelin membrane lipids in model membranes. Biophys J.

[100] Simons K, Ikonen E (1997). Functional rafts in cell membranes. Nature.

[101] Pinto SN, Silva LC, Futerman AH, Prieto M (2011). Effect of ceramide structure on membrane biophysical properties: the role of acyl chain length and unsaturation. Biochim Biophys Acta.

[102] Ibarguren M, López DJ, Encinar JA (2013). Partitioning of liquid-ordered/liquid-disordered membrane microdomains induced by the fluidifying effect of 2-hydroxylated fatty acid derivatives. Biochim Biophys Acta.

[103] Marbois BN, Faull KF, Fluharty AL (2000). Analysis of sulfatide from rat cerebellum and multiple sclerosis white matter by negative ion electrospray mass spectrometry. Biochim Biophys Acta.

[104] Imgrund S, Hartmann D, Farwanah H (2009). Adult ceramide synthase 2 (CERS2)-deficient mice exhibit myelin sheath defects, cerebellar degeneration, and hepatocarcinomas. J Biol Chem.

[105] Pinto SN, Silva LC, de Almeida RFM, Prieto M (2008). Membrane domain formation, interdigitation, and morphological alterations induced by the very long chain asymmetric C24:1 ceramide. Biophys J.

[106] Boggs JM, Gao W, Hirahara Y (2008). Myelin glycosphingolipids, galactosylceramide and sulfatide, participate in carbohydrate-carbohydrate interactions between apposed membranes and may form glycosynapses between oligodendrocyte and/or myelin membranes. Biochim Biophys Acta.

[107] Simons K, Gerl MJ (2010). Revitalizing membrane rafts: new tools and insights. Nat Rev Mol Cell Biol.

[108] DeBruin LS, Haines JD, Wellhauser LA (2005). Developmental partitioning of myelin basic protein into membrane microdomains. J Neurosci Res.

[109] Debruin LS, Harauz G (2007). White matter rafting—membrane microdomains in myelin. Neurochem Res.

[110] Marta CB, Taylor CM, Coetzee T (2003). Antibody cross-linking of myelin oligodendrocyte glycoprotein leads to its rapid repartitioning into detergent-insoluble fractions, and altered protein phosphorylation and cell morphology. J Neurosci.

[111] Kim T, Fiedler K, Madison DL (1995). Cloning and characterization of MVP17: a developmentally regulated myelin protein in oligodendrocytes. J Neurosci Res.

[112] Fitzner D, Schneider A, Kippert A (2006). Myelin basic protein-dependent plasma membrane reorganization in the formation of myelin. EMBO J.

[113] Baron W, Bijlard M, Nomden A (2014). Sulfatide-mediated control of extracellular matrix-dependent oligodendrocyte maturation. Glia.

[114] Park J, Koito H, Li J, Han A (2012). Multi-compartment neuron-glia co-culture platform for localized CNS axon-glia interaction study. Lab Chip.

[115] Liazoghli D, Roth AD, Thostrup P, Colman DR (2012). Substrate micropatterning as a new in vitro cell culture system to study myelination. ACS Chem Neurosci.

[116] Richter-Landsberg C, Heinrich M (1996). OLN-93: a new permanent oligodendroglia cell line derived from primary rat brain glial cultures. J Neurosci Res.

[117] Jung M, Krämer E, Grzenkowski M (1995). Lines of murine oligodendroglial precursor cells immortalized by an activated neu tyrosine kinase show distinct degrees of interaction with axons in vitro and in vivo. Eur J Neurosci.

[118] Kahya N, Schwille P (2006). Fluorescence correlation studies of lipid domains in model membranes. Mol Membr Biol.

[119] Kahya N, Scherfeld D, Bacia K, Schwille P (2004). Lipid domain formation and dynamics in giant unilamellar vesicles explored by fluorescence correlation spectroscopy. J Struct Biol.

[120] Kahya N, Schwille P (2006). How phospholipid–cholesterol interactions modulate lipid lateral diffusion, as revealed by fluorescence correlation spectroscopy. J Fluoresc.

[121] Veatch SL, Keller SL (2005). Miscibility phase diagrams of giant vesicles containing sphingomyelin. Phys Rev Lett.

[122] Kahya N, Scherfeld D, Schwille P (2005). Differential lipid packing abilities and dynamics in giant unilamellar vesicles composed of short-chain saturated glycerol-phospholipids, sphingomyelin and cholesterol. Chem Phys Lipids.

[123] Saadat L, Dupree JL, Kilkus J (2010). Absence of oligodendroglial glucosylceramide synthesis does not result in CNS myelin abnormalities or alter the dysmyelinating phenotype of CGT-deficient mice. Glia.

[124] Bernardino de la Serna J, Perez-Gil J, Simonsen AC, Bagatolli LA (2004). Cholesterol rules: direct observation of the coexistence of two fluid phases in native pulmonary surfactant membranes at physiological temperatures. J Biol Chem.

[125] Dietrich C, Bagatolli LA, Volovyk ZN (2001). Lipid rafts reconstituted in model membranes. Biophys J.

[126] Ruan Q, Cheng MA, Levi M (2004). Spatial-temporal studies of membrane dynamics: scanning fluorescence correlation spectroscopy (SFCS). Biophys J.

[127] Vyas KA, Patel HV, Vyas AA, Schnaar RL (2001). Segregation of gangliosides GM1 and GD3 on cell membranes, isolated membrane rafts, and defined supported lipid monolayers. Biol Chem.

[128] Bagatolli LA (2006). To see or not to see: lateral organization of biological membranes and fluorescence microscopy. Biochim Biophys Acta.

[129] Kahya N (2010). Light on fluorescent lipids in rafts: a lesson from model membranes. Biochem J.

[130] Kahya N, Pécheur EI, de Boeij WP (2001). Reconstitution of membrane proteins into giant unilamellar vesicles via peptide-induced fusion. Biophys J.

[131] Dezi M, Di Cicco A, Bassereau P, Lévy D (2013). Detergent-mediated incorporation of transmembrane proteins in giant unilamellar vesicles with controlled physiological contents. Proc Natl Acad Sci USA.

[132] Girard P, Pécréaux J, Lenoir G (2004). A new method for the reconstitution of membrane proteins into giant unilamellar vesicles. Biophys J.

[133] Boggs JM, Gao W, Zhao J (2010). Participation of galactosylceramide and sulfatide in glycosynapses between oligodendrocyte or myelin membranes. FEBS Lett.

[134] Boggs JM, Wang H (2001). Effect of liposomes containing cerebroside and cerebroside sulfate on cytoskeleton of cultured oligodendrocytes. J Neurosci Res.

[135] Bakhti M, Snaidero N, Schneider D (2013). Loss of electrostatic cell-surface repulsion mediates myelin membrane adhesion and compaction in the central nervous system. Proc Natl Acad Sci USA.

[136] Kippert A, Trajkovic K, Rajendran L (2007). Rho regulates membrane transport in the endocytic pathway to control plasma membrane specialization in oligodendroglial cells. J Neurosci.

[137] Kwiatek JM, Owen DM, Abu-Siniyeh A (2013). Characterization of a new series of fluorescent probes for imaging membrane order. PLoS ONE.

[138] Sanchez SA, Tricerri MA, Gratton E (2012). Laurdan generalized polarization fluctuations measures membrane packing micro-heterogeneity in vivo. Proc Natl Acad Sci USA.

[139] Kim HM, Choo H-J, Jung S-Y (2007). A two-photon fluorescent probe for lipid raft imaging: C-laurdan. Chem Biochem.

[140] Bagatolli LA (2003). Direct observation of lipid domains in free standing bilayers: from simple to complex lipid mixtures. Chem Phys Lipids.

[141] Murugkar S, Brideau C, Ridsdale A (2007). Coherent anti-Stokes Raman scattering microscopy using photonic crystal fiber with two closely lying zero dispersion wavelengths. Opt Express.

[142] Imitola J, Côté D, Rasmussen S (2011). Multimodal coherent anti-Stokes Raman scattering microscopy reveals microglia-associated myelin and axonal dysfunction in multiple sclerosis-like lesions in mice. J Biomed Opt.

[143] Wang H, Fu Y, Zickmund P (2005). Coherent anti-Stokes Raman scattering imaging of axonal myelin in live spinal tissues. Biophys J.

[144] Ries J, Schwille P (2008). New concepts for fluorescence correlation spectroscopy on membranes. Phys Chem Chem Phys.

[145] Philip F, Sengupta P, Scarlata S (2007). Signaling through a G protein-coupled receptor and its corresponding G protein follows a stoichiometrically limited model. J Biol Chem.

[146] Kenworthy AK, Nichols BJ, Remmert CL (2004). Dynamics of putative raft-associated proteins at the cell surface. J Cell Biol.

[147] Pralle A, Keller P, Florin E-L (2000). Sphingolipid–cholesterol rafts diffuse as small entities in the plasma membrane of mammalian cells. J Cell Biol.

[148] Kattnig DR, Bund T, Boggs JM (2012). Lateral self-assembly of 18.5-kDa myelin basic protein (MBP) charge component-C1 on membranes. Biochim Biophys Acta.

[149] Boggs JM, Rangaraj G, Dicko A (2012). Effect of phosphorylation of phosphatidylinositol on myelin basic protein-mediated binding of actin filaments to lipid bilayers in vitro. Biochim Biophys Acta.

[150] Min Y, Alig TF, Lee DW (2011). Critical and off-critical miscibility transitions in model extracellular and cytoplasmic myelin lipid monolayers. Biophys J.

[151] Boggs JM, Rangaraj G, Gao W, Heng Y-M (2006). Effect of phosphorylation of myelin basic protein by MAPK on its interactions with actin and actin binding to a lipid membrane in vitro. Biochemistry.

[152] Boggs JM, Moscarello MA, Papahadjopoulos D (1977). Phase separation of acidic and neutral phospholipids induced by human myelin basic protein. Biochemistry.

[153] Muruganandam G, Bürck J, Ulrich AS (2013). Lipid membrane association of myelin proteins and peptide segments studied by oriented and synchrotron radiation circular dichroism spectroscopy. J Phys Chem B.

[154] Ohler B (2001). Atomic force microscopy of nonhydroxy galactocerebroside nanotubes and their self-assembly at the air–water interface, with applications to myelin. J Struct Biol.

[155] Boggs JM, Yip PM, Rangaraj G, Jo E (1997). Effect of posttranslational modifications to myelin basic protein on its ability to aggregate acidic lipid vesicles†. Biochemistry.

[156] Kim JK, Mastronadi FG, Wood DD (2003). Multiple sclerosis: an important role for post-translational modifications of myelin basic protein in pathogenesis. Mol Cell Proteom.

[157] Wood DD, Moscarello MA (1989). The isolation, characterization, and lipid-aggregating properties of a citrulline containing myelin basic protein. J Biol Chem.

[158] Ahmed MAM, Bamm VV, Harauz G, Ladizhansky V (2010). Solid-state NMR spectroscopy of membrane-associated myelin basic protein–conformation and dynamics of an immunodominant epitope. Biophys J.

[159] Zhong L, Bamm VV, Ahmed MAM (2007). Solid-state NMR spectroscopy of 18.5 kDa myelin basic protein reconstituted with lipid vesicles: spectroscopic characterisation and spectral assignments of solvent-exposed protein fragments. Biochim Biophys Acta.

[160] Frederick KK, Michaelis VK, Corzilius B (2015). Sensitivity-enhanced NMR reveals alterations in protein structure by cellular milieus. Cell.

[161] Musse AA, Gao W, Homchaudhuri L (2008). Myelin basic protein as a “PI(4,5)P2-modulin”: a new biological function for a major central nervous system protein. Biochemistry.

[162] Musse AA, Gao W, Rangaraj G (2009). Myelin basic protein co-distributes with other PI(4,5)P2-sequestering proteins in Triton X-100 detergent-resistant membrane microdomains. Neurosci Lett.

[163] Taraska JW, Zagotta WN (2010). Fluorescence applications in molecular neurobiology. Neuron.

[164] Nawaz S, Kippert A, Saab AS (2009). Phosphatidylinositol 4,5-bisphosphate-dependent interaction of myelin basic protein with the plasma membrane in oligodendroglial cells and its rapid perturbation by elevated calcium. J Neurosci.

[165] Digman MA, Gratton E (2009). Fluorescence correlation spectroscopy and fluorescence cross-correlation spectroscopy. Wiley Interdiscip Rev Syst Biol Med.

[166] Vassall KA, Jenkins AD, Bamm VV, Harauz G (2015). Thermodynamic analysis of the disorder-to-α-helical transition of 18.5-kDa myelin basic protein reveals an equilibrium intermediate representing the most compact conformation. J Mol Biol.

[167] Ishii A, Dutta R, Wark GM (2009). Human myelin proteome and comparative analysis with mouse myelin. Proc Natl Acad Sci USA.

[168] Moscarello MA, Wood DD, Ackerley C, Boulias C (1994). Myelin in multiple sclerosis is developmentally immature. J Clin Invest.

[169] Bradford CM, Ramos I, Cross AK (2014). Localisation of citrullinated proteins in normal appearing white matter and lesions in the central nervous system in multiple sclerosis. J Neuroimmunol.

[170] Hanrieder J, Malmberg P, Ewing AG (2015). Spatial neuroproteomics using imaging mass spectrometry. Biochim Biophys Acta.

[171] Nicklay JJ, Harris GA, Schey KL, Caprioli RM (2013). MALDI imaging and in situ identification of integral membrane proteins from rat brain tissue sections. Anal Chem.

[172] Boggs JM, Moscarello MA (1980). A comparison of composition and fluidity of multiple sclerosis and normal myelin. Neurochem Res.

[173] Saher G, Brügger B, Lappe-Siefke C (2005). High cholesterol level is essential for myelin membrane growth. Nat Neurosci.

[174] Salzer JL (2003). Polarized domains of myelinated axons. Neuron.

[175] Dyer CA, Benjamins JA (1988). Antibody to galactocerebroside alters organization of oligodendroglial membrane sheets in culture. J Neurosci.

[176] Dyer CA, Benjamins JA (1990). Glycolipids and transmembrane signaling: antibodies to galactocerebroside cause an influx of calcium in oligodendrocytes. J Cell Biol.

[177] Dupree JL, Coetzee T, Blight A (1998). Myelin galactolipids are essential for proper node of Ranvier formation in the CNS. J Neurosci.

[178] Dupree JL, Girault JA, Popko B (1999). Axo-glial interactions regulate the localization of axonal paranodal proteins. J Cell Biol.

[179] Bosio A, Binczek E, Stoffel W (1996). Functional breakdown of the lipid bilayer of the myelin membrane in central and peripheral nervous system by disrupted galactocerebroside synthesis. Proc Natl Acad Sci USA.

[180] Ishibashi T, Dupree JL, Ikenaka K (2002). A myelin galactolipid, sulfatide, is essential for maintenance of ion channels on myelinated axon but not essential for initial cluster formation. J Neurosci.

[181] Schafer DP, Bansal R, Hedstrom KL (2004). Does paranode formation and maintenance require partitioning of neurofascin 155 into lipid rafts?. J Neurosci.

[182] Gielen E, Vercammen J, Sýkora J (2005). Diffusion of sphingomyelin and myelin oligodendrocyte glycoprotein in the membrane of OLN-93 oligodendroglial cells studied by fluorescence correlation spectroscopy. C R Biol.

[183] Gielen E, Smisdom N, De Clercq B (2008). Diffusion of myelin oligodendrocyte glycoprotein in living OLN-93 cells investigated by raster-scanning image correlation spectroscopy (RICS). J Fluoresc.

[184] Gielen E, Smisdom N, vandeVen M (2009). Measuring diffusion of lipid-like probes in artificial and natural membranes by raster image correlation spectroscopy (RICS): use of a commercial laser-scanning microscope with analog detection. Langmuir.

